# Chronic Obstructive Pulmonary Disease and the Optimal Timing of Lung Transplantation

**DOI:** 10.3390/medicina55100646

**Published:** 2019-09-26

**Authors:** Rodrigo Vazquez Guillamet

**Affiliations:** School of Medicine, Washington University, St. Louis, MO 63130, USA; r.vazquezguillamet@wustl.edu

**Keywords:** lung transplant, chronic obstructive pulmonary disease, wait list, survival

## Abstract

Chronic obstructive pulmonary disease (COPD) accounts for the largest proportion of respiratory deaths worldwide and was historically the leading indication for lung transplantation. The success of lung transplantation procedures is measured as survival benefit, calculated as survival with transplantation minus predicted survival without transplantation. In chronic obstructive pulmonary disease, it is difficult to show a clear and consistent survival benefit. Increasing knowledge of the risk factors, phenotypical heterogeneity, systemic manifestations, and their management helps improve our ability to select candidates and list those that will benefit the most from the procedure.

## Clinical Vignette:

A 64-year-old female with 50 pack years of smoking presented for lung transplant evaluation; she had quit smoking for two years. Her forced expiratory volume in the first second percent predicted (FEV_1_%) was 25% and her body mass index, airflow obstruction, dyspnea, and exercise capacity (BODE) index was 6. She had suffered a severe exacerbation, which required non-invasive mechanical ventilation three months prior to presentation. Past medical history included asthma as a child, but no other comorbidities. For the last eight years, she has been on oxygen 24/7, and has limited her activities outside her household—first, out of embarrassment and over the last two years because of generalized weakness and severe dyspnea. Her main goals after transplantation were to be able to go fishing with her husband and be independent again. The patient underwent transplantation and was able to meet her stated goals. Unfortunately, and despite a pre-transplantation coronary angiography with minimal luminal stenosis, she experienced sudden cardiac death from a myocardial infarction 20 months after the procedure.

## 1. General Concepts in Lung Transplantation

### 1.1. Timing for Listing in Lung Transplantation

The optimal timing for listing in lung transplantation maximizes survival and quality of life [1]. Demonstrating a survival advantage for patients with chronic obstructive pulmonary disease (COPD) has been difficult and improvement in quality of life has not been thoroughly studied [2]. In the next paragraphs, we will discuss the factors impacting timing for transplantation, while reviewing the epidemiology of COPD, along with new advancements in understanding this disease. We will also focus on how comorbidities and lung reduction procedures may impact transplantation timing.

### 1.2. New Lung Allocation Systems Have Decreased the Proportion of Transplants Performed for End-Stage Chronic Obstructive Pulmonary Disease

COPD contributes significantly to respiratory deaths worldwide [3] and is caused by exposure to noxious particles, fumes, and gases, resulting in different degrees of airway wall thickening, lumen obstruction by mucus (chronic bronchitis), and loss of alveolar walls (emphysema) [4,5]. In a particular patient, COPD can present as single disease or can coexist with other airway and parenchymal processes [6]. In its most advanced stages, it leads to respiratory failure and death [3].

Patients with end-stage COPD can be considered as candidates for lung transplantation; traditionally, this disease has been the most common indication for lung transplantation [7]. Both higher prevalence compared to other respiratory diseases, as well as lung allocation systems prioritizing transplant based on timed accrued on the list [8], contributed to this ranking. Under those circumstances, COPD patients had a survival advantage over other end-stage lung diseases, which resulted in a lower mortality on the waiting list and a higher probability of transplantation.

The introduction of new allocation systems in 2005 changed the outlook of transplantation [8,9]. These new policies attempted to decrease waitlist mortality by prioritizing transplantation based on urgency and survival benefit. Because the survival benefit in interstitial lung diseases (ILDs) is clearer, they now comprise approximately 50% of lung transplantation procedures, while being responsible for only 0.2% of all deaths and 0.1% of all disability adjusted life years (DALYs) per 100,000 inhabitants worldwide. Meanwhile, COPD now accounts for 30% of the procedures while causing 5.7% of all deaths and accounting for 3.3% DALYs per 100,000 inhabitants worldwide [3] (28 and 33 times higher than interstitial lung diseases). Development of better mortality prediction systems for COPD, as well as the inclusion of quality of life measures in future allocation systems, have the potential to mitigate this trend.

### 1.3. Survival Benefit in Lung Transplantation

It is calculated as the expected survival after undergoing the transplantation surgery minus expected survival with maximal medical/surgical therapy exclusive of transplantation [10]. Any factors that alter any of the two components of this equation will influence the appropriate timing of transplantation.

### 1.4. Current Listing Practices 

Patients with COPD are enrolled on the lung transplantation list when the following conditions are met: (1) COPD is severe and expected to cause death within the next two years with a high probability (>50%). (2) The probability of comorbidity-related mortality at 1 and 5 years if the lung allograft is still functional is <20%.

## 2. Natural History of COPD

Gaps in the knowledge of the natural history of COPD affect our ability to better time lung transplantation. First of all, the inciting events that start the process have not been clearly defined [11]. There is increasing evidence that epigenetic changes [12,13] can have an effect on the development of COPD. The maximal lung function attained also impacts the chances of COPD-related disability later in life [14]. Furthermore, once established, not everyone progresses in a similar fashion, with some patients surviving for many years, even in the most advanced stages of the disease, while others decline quickly through the final stages of the disease [15,16].

COPD can co-exist with other respiratory conditions that affect the risk of progression towards respiratory failure. Lung function [17] declines more slowly in asthma-COPD overlap patients. On the other hand, the co-existence of interstitial lung disease with COPD appears to accelerate the process [18]. The effect of chronic bronchitis or the coexistence of bronchiectasis has not been clearly defined.

COPD also behaves as a systemic disease. COPD patients have a higher prevalence of coronary artery disease; congestive heart failure; esophageal, breast, pancreatic and lung cancer; diabetes mellitus; cirrhosis; and peptic ulcer disease [19,20]. COPD patients on the transplant waiting list also suffer from higher rates of osteoporosis than interstitial lung disease and pulmonary hypertension candidates due to chronic exposure to steroids, malnutrition, and immobility [21]. Finally, emphysematous patients in particular are at a higher risk of developing abdominal hernias and aneurysms [22].

A key assumption of the urgency allocation systems is that we can clearly predict survival and transplantation benefit for each individual patient. However, as outlined above, COPD is more a syndrome than a disease, and the combinations of predisposing factors, comorbidities, and coexisting respiratory diseases result in a different risk of death and complications after lung transplantation. A continued effort to refine mortality prediction rules is needed.

### Predicting Mortality in COPD

Extensive literature supports the role of spirometry in predicting mortality. Different measures, including the forced expiratory volume in one second (FEV_1_%), forced vital capacity (FVC) and their ratio (FEV_1_/FVC) have been used with prognostic purposes [17,23]. FEV_1_% predicted <15% or 20% is used as one of the criteria for listing in the International Society for Heart and Lung Transplantation (ISHLT) consensus statement [1]. Patients with COPD can die from respiratory complications, but also from cardiovascular disease and malignancies [24]. This criterion for listing is more specific for respiratory deaths, which are the ones we can expect to prevent through lung transplantation. The disadvantage is that although patients have an increased mortality, a low FEV_1_% does not preclude prolonged survival. The high-risk group of the National Emphysema Treatment Trial defined by FEV_1_% between 15% and 20%, residual volume (RV) > 150% predicted, and low exercise capacity had a mortality of 51.4% at the mean follow up of 29.2 months [15].

Other pulmonary function indices of airflow obstruction are predictors of mortality, including RV, total lung capacity (TLC), and RV/TLC ratio. However, they are less studied and rarely measured in clinical practice [25,26,27].

Measures of gas transfer, carbon monoxide diffusing capacity (DLCO), and arterial partial pressure of oxygen (PaO_2_) have also been explored as predictors of mortality. In a cohort of 604 patients from the United Kingdom, they were, along with age, the only predictors for mortality in multivariable models eclipsing FEV_1_% predicted and other measures of obstruction. It is worth noting that the median survival in the lowest quartile of DLCO—defined as <27.9—was around 60 months, far better than the 50% (two years) probability of death required to be considered for lung transplantation [28].

Severe exacerbations of COPD are variably defined in the literature [29]. They are, however, invariably associated with increased mortality [30,31]. Patients who survive to discharge after being admitted to the hospital for a COPD exacerbation have a two-year mortality between 20% for general floor admission and 50% for patients with hypercapnic respiratory failure or need for mechanical ventilation. A history of severe exacerbation, defined as one needing hospital care (admission or ED visit), also increases the chances of dying from a respiratory versus other-than-respiratory death (e.g., cardiovascular and malignancy related deaths) [24]. When there are no modifiable treatment factors, including environmental exposures and medical treatment, this group becomes particularly attractive for enrollment in the wait list.

Finally, the ISLHT consensus document includes the co-existence of pulmonary hypertension as an indication to list patients for transplantation [1]. COPD accounts for 80–90% of cases of WHO group III pulmonary hypertension. Chronic hypoxemia leads to vascular remodeling and increased pulmonary vascular resistance. A French group found a prevalence of severe pulmonary hypertension (defined as pulmonary artery pressure > 40 mmHg of 3%) in a cohort of patients recently discharged from the hospital after treatment for acute exacerbations of COPD. Approximately 50% of them were dead at two years. Although half of them also had comorbid conditions that could explain/contribute to the presence of pulmonary arterial hypertension and could be considered as absolute or relative contraindications to lung transplantation, including obesity hypoventilation syndrome, left ventricular dysfunction and connective tissue disease, the other half did not and may have benefitted from transplantation [32].

Research efforts in recent years have focused on frailty, which represents another factor associated with wait list mortality in patients with COPD. Frailty is defined as a state of low physiological reserve and increased susceptibility to insults [33]. Frail patients on the lung transplant list are at a higher risk of being delisted and dying prior to surgery [33]. They have also been found to have a higher risk of post-transplant death [34]. A recent study described the trajectories of frail patients comparing pre-and post-transplant reports. Interestingly, 84% of 51 patients (with any diagnosis) considered frail prior to transplantation became non-frail six months after transplant [35].

Realizing the limitations of individual variables for mortality prediction for such a heterogenous disease, the field has moved towards multi-dimensional tools that aim at accounting both the pulmonary and extra pulmonary manifestations of the disease. The most widely used are the Body Mass Index (BMI), Obstruction, Dyspnea and Exercise Capacity (BODE) [36] and the Age, Dyspnea and Obstruction (ADO) indices [31]. The former is one of the criteria recommended by the ISHLT to assess the severity of the disease and appropriate timing for listing. In the original and validation cohorts, a BODE score > 7 was associated with a probability of mortality of 50% at 3 years and 80% at 4 years. Using the SRTR database and the survival predictions based on the original BODE cohort, it was estimated that patients with a BODE index > 7 would benefit from a longer median survival after transplantation [37]. One limitation of this study was that the derivation and validation cohorts for the BODE index were general pulmonary populations, whereas the patients listed for lung transplantation are highly selected and experience lower mortality rates. This was highlighted in another report, which found a median survival of 59 months (95% CI, 51–77 months) in the UNOS cohort and 37 months in the BODE validation cohort [38]. In chronic stable severe COPD, an ADO index of ≥7 is associated with 47.2% mortality at 3 years. The ADO score has not been validated for use in patients awaiting lung transplantation [39].

Thabut and colleagues evaluated COPD patients undergoing transplantation between 1987 and 2004. Age, functional status, alpha 1 antitrypsin deficiency, hypoxemia at rest, six-minute walk distance, continuous mechanical ventilation, pulmonary artery hypertension, and BMI influenced mortality [2]. Updated versions of their equations might be better suited for use in clinical practice and for the particular purpose of predicting mortality on the lung transplantation wait list.

## 3. Relative Contraindications’ Impact on the Timing for Transplantation

As a general rule, a contraindication is relative when interventions can be implemented that will minimize its effect on survival. Instauration of such measures may delay transplantation. The “comorbidome” described in COPD includes comorbidities that are disproportionally diagnosed in COPD patients: osteoporosis; coronary artery disease; congestive heart failure; esophageal, breast, pancreatic, and lung cancer; diabetes mellitus; cirrhosis; and peptic ulcer disease [19]. These diagnoses are generally considered absolute or relative contraindications to transplantation when they are not properly controlled.

Osteoporosis is particularly prevalent in patients with end-stage COPD and is found in 59 to 69% of patients prior to transplantation [40,41]. It is diagnosed when bone mineral density is 2.5 standard deviations below average (T-score is equal of less than −2.5) and worsens significantly after transplantation. Osteoporotic patients have an increased risk for fractures that can be life threatening in the postoperative period and affect quality of life and lung function later on [40,42]. If time allows, the main interventions to prevent fractures that should be implemented are minimizing steroid use before transplantation, an adequate diet to maintain a body mass index above 21 kg/m^2^, daily exercise training, avoiding alcohol, and the use of pharmacological therapy, mainly bisphosphonates along with calcium and vitamin D supplementation [43]. Temporal trends in the published literature suggest that these interventions may decrease the incidence of fractures from 40–50% in early cohorts to 5% to 15% in current studies; however, the role of other contributing factors could not be ruled out [21,44,45]. If osteoporosis is severe enough that life-threatening fractures have already occurred or is present along with other comorbidities, withholding transplantation should be considered.

Coronary artery disease (CAD) is another important comorbidity [46]. In the last five years, several groups have reported comparisons of the outcomes of patients with CAD prior to transplantation and any end-stage lung disease. Although it is difficult to compare their results owing to varying definitions and severity of disease, overall lung transplantation is possible in patients and has similar outcomes with two caveats: patients requiring revascularization had longer hospital stays and patients with at least 50% luminal stenosis had a higher incidence of cardiac events post-transplant [47,48]. The best method for revascularization should be individualized and should follow standard international guidelines. If percutaneous interventions are performed, it should be kept in mind that the minimum recommended duration of dual antiplatelet therapy is four weeks for bare metal stents and 12 to 24 weeks for drug eluting stents [49].

The landscape of cancer diagnosis and treatment is one of the fastest-evolving fields in medicine. Mortality of patients diagnosed with cancer after transplantation is generally higher than for those diagnosed prior to transplantation [50]. Usually patients should be cancer free for 2 to 5 years prior to listing. This recommendation is based on registry data indicating a decrease in the risk of recurrence from 15% at 3 years to <5% at 5 years [51]. Currently, personalized measures of cancer recurrence risk, tumor-specific clinical calculators, as well as tumor genomic profiling tools [52] can be employed and may impact the timing of transplantation as more experience accumulates.

Liver cirrhosis is in general a contraindication to lung transplantation. Although consideration has been given to lung-liver transplantation, the procedure is rarely performed with a total of 116 recipients from 1994 to 9/2019 [53]. A particular group of patients of interest in COPD are patients with alpha 1 antitrypsin deficiency. In rare occasions, they present with simultaneous advanced lung and liver disease. The experience in this subset of patients amounts to case reports [54,55] and the selection and timing of multi-organ transplantation is beyond the scope of this review.

Finally, diabetes mellitus (DM) and peptic ulcer disease are relative and not considered contraindications to lung transplantation; they can usually be optimized prior to transplantation. In the case of DM, the presence of end organ damage is the main concern. Peptic ulcer disease should be treated and if the risk of bleeding is high, transplantation should be delayed.

## 4. Lung Volume Reduction Surgery and Bronchoscopic Volume Reduction

In the National Emphysema Treatment Trial, which compared surgical lung volume reduction surgery to maximal medical treatment, patients with upper lobe predominant emphysema, hyperinflation, and low exercise capacity experienced improvements in lung function and survival after lung volume reduction surgery (LVRS) [15]. Although previous thoracotomy increases the risk of perioperative complications, the risk is not prohibitive and a history of the procedure is not a contraindication to undergoing lung transplantation. Early cohorts, including an analysis of the UNOS registry, suggested similar survival after transplantation; however, more recent cohorts report an increase in the incidence of perioperative complications, lower long-term survival and higher incidence of chronic rejection [56,57,58,59].

Bronchoscopic lung volume reduction is an alternative to surgical treatment. The procedure is relatively simple and performed using one-way valves, adhesive material, or coils that effectively deflate the most affected areas of the lung. Possible advantages to patients being considered for transplantation include preserving an intact pleural space with improved lung function and exercise capacity, which may result in improvement in fitness for transplantation [60]. A major drawback is the relatively high incidence of exacerbations after the procedure with inherent risks [61].

## 5. Conclusions

The patient in the introductory clinical vignette achieved all her goals from the procedure: she became independent and was able to go fishing with her husband. However, she died of a myocardial infarction, despite a normal coronary angiography without a survival benefit. The inability of current screening methods to predict her poor cardiovascular outcome highlights the need to further our knowledge on how to better characterize this heterogeneous disease, its natural history, and its complications. Increasing knowledge in all these areas will help transplant physicians time lung transplantation better.

## References

[1] Weill D., Benden C., Corris P.A., Dark J.H., Davis R.D., Keshavjee S., Lederer D.J., Mulligan M.J., Patterson G.A., Singer L.G. (2015). A consensus document for the selection of lung transplant candidates: 2014—An update from the Pulmonary Transplantation Council of the International Society for Heart and Lung Transplantation. J. Heart Lung Transplant..

[2] Thabut G., Ravaud P., Christie J.D., Castier Y., Fournier M., Mal H., Lesèche G., Porcher R. (2008). Determinants of the survival benefit of lung transplantation in patients with chronic obstructive pulmonary disease. Am. J. Respir. Crit. Care Med..

[3] GBD Compare IHME Viz Hub. http://vizhub.healthdata.org/gbd-compare.

[4] Galban C., Chamberlain R., Hoff B., Johnson T., Kazerooni E., Martinez F., Ross B., Lynchg D., Han M. (2015). Parametric response mapping of COPD phenotypes: A COPDGene study. Eur. Respir. J..

[5] MacNee W. (2006). Pathology, pathogenesis, and pathophysiology. BMJ.

[6] Pavord I.D., Wardlaw A.J. (2010). The A to E of airway disease. Clin. Exp. Allergy.

[7] ISHLT: The International Society for Heart & Lung Transplantation. https://ishltregistries.org/registries/slides.asp.

[8] Valapour M., Lehr C.J., Skeans M.A., Smith J.M., Uccellini K., Lehman R., Robinson A., Israni A.K., Snyder J.J., Kasiske B.L. (2019). OPTN/SRTR 2017 Annual Data Report: Lung. Am. J. Transplant..

[9] Liu V., Zamora M.R., Dhillon G.S., Weill D. (2010). Increasing Lung Allocation Scores predict worsened survival among lung transplant recipients. Am. J. Transplant..

[10] Vock D.M., Durheim M.T., Tsuang W.M., Copeland C.A.F., Tsiatis A.A., Davidian M., Neely M.L., Lederer D.J., Palmer S.M. (2017). Survival Benefit of Lung Transplantation in the Modern Era of Lung Allocation. Ann. Am. Thorac. Soc..

[11] Petersen H., Vazquez Guillamet R., Meek P., Sood A., Tesfaigzi Y. (2018). Early Endotyping: A Chance for Intervention in Chronic Obstructive Pulmonary Disease. Am. J. Respir. Cell Mol. Biol..

[12] Beyer D., Mitfessel H., Gillissen A. (2009). Maternal smoking promotes chronic obstructive lung disease in the offspring as adults. Eur. J. Med. Res.

[13] Singh S.P., Gundavarapu S., Peña-Philippides J.C., Rir-sima-ah J., Mishra N.C., Wilder J.A., Langley R.J., Smith K.R., Sopori M.L. (2011). Prenatal Secondhand Cigarette Smoke Promotes Th2 polarization and impairs goblet cell differentiation and airway mucus formation. J. Immunol..

[14] Lange P., Celli B., Agustí A., Boje Jensen G., Divo M., Faner R., Guerra S., Marott J.L., Martinez F.D., Martinez-Camblor P. (2015). Lung-Function Trajectories Leading to Chronic Obstructive Pulmonary Disease. N. Engl. J. Med..

[15] Fishman A., Martinez F., Naunheim K., Piantadosi S., Wise R., Ries A., Weinmann G., Wood D.E. (2003). National Emphysema Treatment Trial Research Group A randomized trial comparing lung-volume-reduction surgery with medical therapy for severe emphysema. N. Engl. J. Med..

[16] Sood A., Petersen H., Qualls C., Meek P.M., Vazquez-Guillamet R., Celli B.R., Tesfaigzi Y. (2016). Spirometric variability in smokers: Transitions in COPD diagnosis in a five-year longitudinal study. Respir. Res..

[17] Fortis S., Comellas A., Make B.J., Hersh C.P., Bodduluri S., Georgopoulos D., Kim V., Criner G.J., Dransfield M.T., Bhatt S.P. (2019). Combined FEV1 and FVC Bronchodilator Response, Exacerbations, and Mortality in COPD. Ann. Am. Thorac. Soc..

[18] Jacob J., Bartholmai B.J., Rajagopalan S., Kokosi M., Maher T.M., Nair A., Karwoski R., Renzoni E., Walsh S.L.F., Hansell D.M. (2017). Functional and prognostic effects when emphysema complicates idiopathic pulmonary fibrosis. Eur. Respir. J..

[19] Divo M., Cote C., de Torres J.P., Casanova C., Marin J.M., Pinto-Plata V., Zulueta J., Cabrera C., Zagaceta J., Hunninghake G. (2012). Comorbidities and Risk of Mortality in Patients with Chronic Obstructive Pulmonary Disease. Am. J. Respir Crit Care Med..

[20] Baty F., Putora P.M., Isenring B., Blum T., Brutsche M. (2013). Comorbidities and burden of COPD: A population based case-control study. PLoS ONE.

[21] Spira A., Gutierrez C., Chaparro C., Hutcheon M.A., Chan C.K.N. (2000). Osteoporosis and Lung Transplantation: A Prospective Study. Chest.

[22] Akturk U.A., Kocak N.D., Akturk S., Dumantepe M., Sengul A., Akcay M.A., Akbay M.O., Kabadayi F., Ernam D. (2017). What are the Prevalence of Abdominal Aortic Aneurysm in Patients with Chronic Obstructive Pulmonary Diseases and the Characteristics of These Patients?. Eurasian J. Med..

[23] Almagro P., Martinez-Camblor P., Soriano J.B., Marin J.M., Alfageme I., Casanova C., Esteban C., Soler-Cataluña J.J., de-Torres J.P., Celli B.R. (2014). Finding the Best Thresholds of FEV1 and Dyspnea to Predict 5-Year Survival in COPD Patients: The COCOMICS Study. PLoS ONE.

[24] Abukhalaf J., Davidson R., Villalobos N., Meek P., Petersen H., Sood A., Tesfaigzi Y., Vazquez Guillamet R. (2018). Chronic obstructive pulmonary disease mortality, a competing risk analysis. Clin. Respir. J..

[25] Shin T.R., Oh Y.-M., Park J.H., Lee K.S., Oh S., Kang D.R., Sheen S., Seo J.B., Yoo K.H., Lee J.-H. (2015). The Prognostic Value of Residual Volume/Total Lung Capacity in Patients with Chronic Obstructive Pulmonary Disease. J. Korean Med. Sci.

[26] Kim Y.W., Lee C.-H., Hwang H.-G., Kim Y.-I., Kim D.K., Oh Y.-M., Lee S.H., Kim K.U., Lee S.-D. (2019). Resting hyperinflation and emphysema on the clinical course of COPD. Sci. Rep..

[27] Han M.K., Kim M.G., Mardon R., Renner P., Sullivan S., Diette G.B., Martinez F.J. (2007). Spirometry utilization for COPD: How do we measure up?. Chest.

[28] Boutou A.K., Shrikrishna D., Tanner R.J., Smith C., Kelly J.L., Ward S.P., Polkey M.I., Hopkinson N.S. (2013). Lung function indices for predicting mortality in COPD. Eur. Respir. J..

[29] Kim V., Aaron S.D. (2018). What is a COPD exacerbation? Current definitions, pitfalls, challenges and opportunities for improvement. Eur. Respir. J..

[30] Hartl S., Lopez-Campos J.L., Pozo-Rodriguez F., Castro-Acosta A., Studnicka M., Kaiser B., Roberts C.M. (2016). Risk of death and readmission of hospital-admitted COPD exacerbations: European COPD Audit. Eur. Respir. J..

[31] Espantoso-Romero M., Román Rodríguez M., Duarte-Pérez A., Gonzálvez-Rey J., Callejas-Cabanillas P.A., Lazic D.K., Anta-Agudo B., Torán Monserrat P., Magallon-Botaya R., Gerasimovska Kitanovska B. (2016). External validation of multidimensional prognostic indices (ADO, BODEx and DOSE) in a primary care international cohort (PROEPOC/COPD cohort). BMC Pulm. Med..

[32] Chaouat A., Bugnet A.-S., Kadaoui N., Schott R., Enache I., Ducoloné A., Ehrhart M., Kessler R., Weitzenblum E. (2005). Severe pulmonary hypertension and chronic obstructive pulmonary disease. Am. J. Respir. Crit. Care Med..

[33] Singer J.P., Diamond J.M., Gries C.J., McDonnough J., Blanc P.D., Shah R., Dean M.Y., Hersh B., Wolters P.J., Tokman S. (2015). Frailty Phenotypes, Disability, and Outcomes in Adult Candidates for Lung Transplantation. Am. J. Respir. Crit. Care Med..

[34] Wilson M.E., Vakil A.P., Kandel P., Undavalli C., Dunlay S.M., Kennedy C.C. (2016). Pretransplant frailty is associated with decreased survival after lung transplantation. J. Heart Lung Transplant..

[35] Venado A., McCulloch C., Greenland J.R., Katz P., Soong A., Shrestha P., Hays S., Golden J., Shah R., Leard L.E. (2019). Frailty trajectories in adult lung transplantation: A cohort study. J. Heart Lung Transplant..

[36] Cote C.G., Pinto-Plata V.M., Marin J.M., Nekach H., Dordelly L.J., Celli B.R. (2008). The modified BODE index: Validation with mortality in COPD. Eur. Respir. J..

[37] Lahzami S., Bridevaux P.O., Soccal P.M., Wellinger J., Robert J.H., Ris H.B., Aubert J.D. (2010). Survival impact of lung transplantation for COPD. Eur. Respir. J..

[38] Reed R.M., Cabral H., Dransfield M., Eberlein M., Merlo C., Mulligan M.J., Netzer G., Sanchez P.G., Scharf S.M., Sin D.D. (2017). Survival of Lung Transplant Candidates with COPD: BODE Score Reconsidered. Chest.

[39] Puhan M.A., Hansel N.N., Sobradillo P., Enright P., Lange P., Hickson D., Menezes A.M., ter Riet G., Held U., Domingo-Salvany A. (2012). Large-scale international validation of the ADO index in subjects with COPD: An individual subject data analysis of 10 cohorts. BMJ Open.

[40] Førli L., Mellbye O.J., Halse J., Bjørtuft O., Vatn M., Boe J. (2008). Cytokines, bone turnover markers and weight change in candidates for lung transplantation. Pulm. Pharmacol. Ther..

[41] Tschopp O., Boehler A., Speich R., Weder W., Seifert B., Russi E.W., Schmid C. (2002). Osteoporosis before lung transplantation: Association with low body mass index, but not with underlying disease. Am. J. Transplant..

[42] Kananen K., Volin L., Laitinen K., Alfthan H., Ruutu T., Välimäki M.J. (2005). Prevention of bone loss after allogeneic stem cell transplantation by calcium, vitamin D, and sex hormone replacement with or without pamidronate. J. Clin. Endocrinol. Metab..

[43] Wagner D., Amrein K., Dimai H.P., Kniepeiss D., Tscheliessnigg K.H., Kornprat P., Dobnig H., Pieber T., Fahrleitner-Pammer A. (2012). Ibandronate and calcitriol reduces fracture risk, reverses bone loss, and normalizes bone turnover after LTX. Transplantation.

[44] Shane E., Papadopoulos A., Staron R.B., Addesso V., Donovan D., McGregor C., Schulman L.L. (1999). Bone loss and fracture after lung transplantation. Transplantation.

[45] Hariman A., Alex C., Heroux A., Camacho P. (2014). Incidence of Fractures after Cardiac and Lung Transplantation: A Single Center Experience. J. Osteoporos..

[46] Reed R.M., Eberlein M., Girgis R.E., Hashmi S., Iacono A., Jones S., Netzer G., Scharf S. (2012). Coronary Artery Disease Is Under-diagnosed and Under-treated in Advanced Lung Disease. Am. J. Med..

[47] Castleberry A.W., Martin J.T., Osho A.A., Hartwig M.G., Hashmi Z.A., Zanotti G., Shaw L.K., Williams J.B., Lin S.S., Davis R.D. (2013). Coronary Revascularization in Lung Transplant Recipients With Concomitant Coronary Artery Disease. Am. J. Transplant..

[48] Sherman W., Rabkin D.G., Ross D., Saggar R., Lynch J.P., Belperio J., Saggar R., Hamilton M., Ardehali A. (2011). Lung transplantation and coronary artery disease. Ann. Thorac. Surg..

[49] Kereiakes D.J., Yeh R.W., Massaro J.M., Driscoll-Shempp P., Cutlip D.E., Steg P.G., Gershlick A.H., Darius H., Meredith I.T., Ormiston J. (2015). Antiplatelet Therapy Duration Following Bare Metal or Drug-Eluting Coronary Stents: The Dual Antiplatelet Therapy Randomized Clinical Trial. JAMA.

[50] Johnson E.E., Leverson G.E., Pirsch J.D., Heise C.P. (2007). A 30-year analysis of colorectal adenocarcinoma in transplant recipients and proposal for altered screening. J. Gastrointest. Surg..

[51] Chapman J.R., Sheil A.G., Disney A.P. (2001). Recurrence of cancer after renal transplantation. Transplant. Proc..

[52] Mukhtar R.A., Piper M.L., Freise C., Veer L.J.V., Baehner F.L., Esserman L.J. (2017). The Novel Application of Genomic Profiling Assays to Shorten Inactive Status for Potential Kidney Transplant Recipients With Breast Cancer. Am. J. Transplant..

[53] National Data - OPTN. https://optn.transplant.hrsa.gov/data/view-data-reports/national-data.

[54] Freischlag K.W., Messina J., Ezekian B., Mulvihill M.S., Barbas A., Berg C., Sudan D., Reynolds J., Hartwig M., Knechtle S. (2018). Single-Center Long-Term Analysis of Combined Liver-Lung Transplant Outcomes. Transplant. Direct..

[55] Yi S.G., Burroughs S.G., Loebe M., Scheinin S., Seethamraju H., Jyothula S., Monsour H., McFadden R., Podder H., Saharia A. (2014). Combined lung and liver transplantation: Analysis of a single-center experience. Liver Transpl..

[56] Nathan S.D., Edwards L.B., Barnett S.D., Ahmad S., Burton N.A. (2004). Outcomes of COPD lung transplant recipients after lung volume reduction surgery. Chest.

[57] Backhus L., Sargent J., Cheng A., Zeliadt S., Wood D., Mulligan M. (2014). Outcomes in lung transplantation after previous lung volume reduction surgery in a contemporary cohort. J. Thorac. Cardiovasc. Surg..

[58] Shigemura N., Gilbert S., Bhama J.K., Crespo M.M., Zaldonis D., Pilewski J.M., Bermudez C.A. (2013). Lung transplantation after lung volume reduction surgery. Transplantation.

[59] Patel N., DeCamp M., Criner G.J. (2008). Lung Transplantation and Lung Volume Reduction Surgery versus Transplantation in Chronic Obstructive Pulmonary Disease. Proc. Am. Thorac Soc..

[60] Venuta F., Diso D., Anile M., De Giacomo T., Rendina E.A., Rolla M., Ricella C., Coloni G.F. (2011). Bronchoscopic lung volume reduction as a bridge to lung transplantation in patients with chronic obstructive pulmonary disease. Eur. J. Cardiothorac. Surg..

[61] Gülşen A. (2018). Bronchoscopic Lung Volume Reduction: A 2018 Review and Update. Turk. Thorac. J..

